# Supplementary material for: Mixed recurrent connectivity in primate prefrontal cortex
Source: PLoS Comput Biol. 2025 Mar 11;21(3):e1012867. doi: 10.1371/journal.pcbi.1012867 (PMC11918408; doi:10.1371/journal.pcbi.1012867)
Supplement: S1 Table — In parenthesis, the number of selective neurons is shown. (DOCX) [file pcbi.1012867.s001.docx]

| **Monkey** | **FEF**  **# of recorded Neurons (# of selective neurons)** | **LPFC**  **# of recorded Neurons**  **(# of selective neurons)** |
| --- | --- | --- |
| J | 54 (21) | 23 (5) |
| P | 5 (3) | 32 (19) |
| W | 73 (37) | 36 (12) |
| **All Monkeys** | **132 (61) - 46%** | **91 (36) - 39%** |

**Table S1**. *Number of neurons recorded in each monkey and each region. In parenthesis, the number of selective neurons is shown*.
