# Supplementary material for: Mixed recurrent connectivity in primate prefrontal cortex
Source: PLoS Comput Biol. 2025 Mar 11;21(3):e1012867. doi: 10.1371/journal.pcbi.1012867 (PMC11918408; doi:10.1371/journal.pcbi.1012867)
Supplement: S2 Table — In parenthesis, the number of significant pairs/neurons is shown. (DOCX) [file pcbi.1012867.s002.docx]

| **FEF**  **Significant Pairs** | **PFC**  **Significant Pairs** | **FEF**  **Significant Neurons** | **LPFC**  **Significant Neurons** |
| --- | --- | --- | --- |
| **41 (15) - 36%** | **30 (3) - 10%** | **48 (21) - 44%** | **17 (5) - 29%** |

**Table S2.** *Number of neuron pairs with significant noise correlation and numbers of neurons in these pairs in FEF and DLPFC (note that one neuron can belong to multiple pairs). In parenthesis, the number of significant pairs/neurons is shown.*
