# Supplementary figures and images for: Mixed recurrent connectivity in primate prefrontal cortex
Source: PLoS Comput Biol. 2025 Mar 11;21(3):e1012867. doi: 10.1371/journal.pcbi.1012867 (PMC11918408; doi:10.1371/journal.pcbi.1012867)

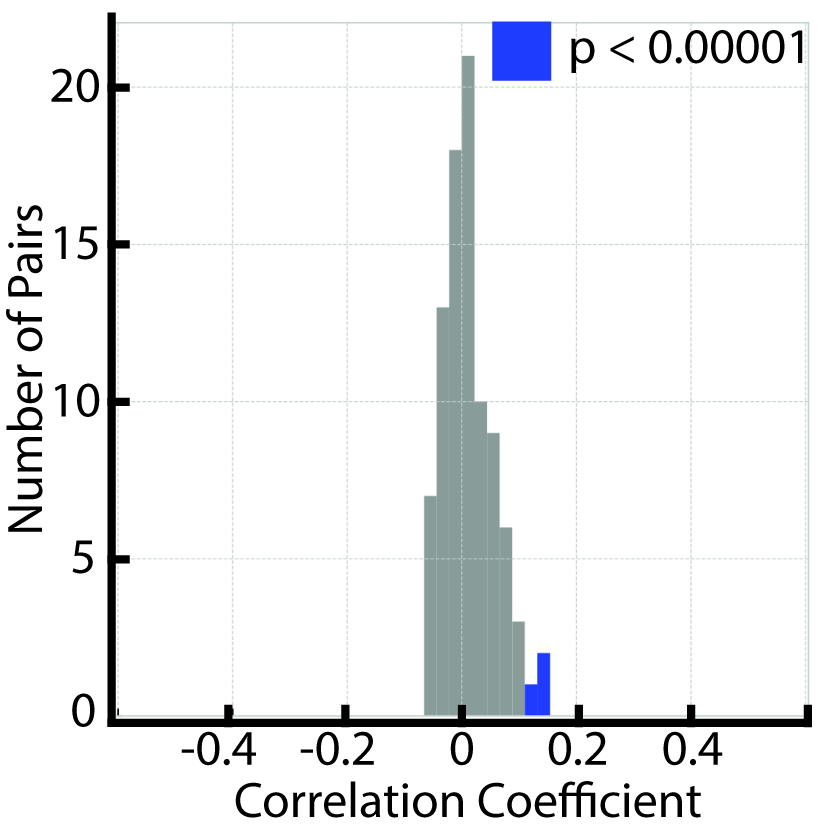

Supplement: S1 Fig — The correlation coefficient for the neuron pairs with overlapping selectivity only for pairs that contain one neuron from FEF and one from DLPFC (blue bars: significant values; gray bars: non-significant values). (TIF) [file pcbi.1012867.s003.tif]

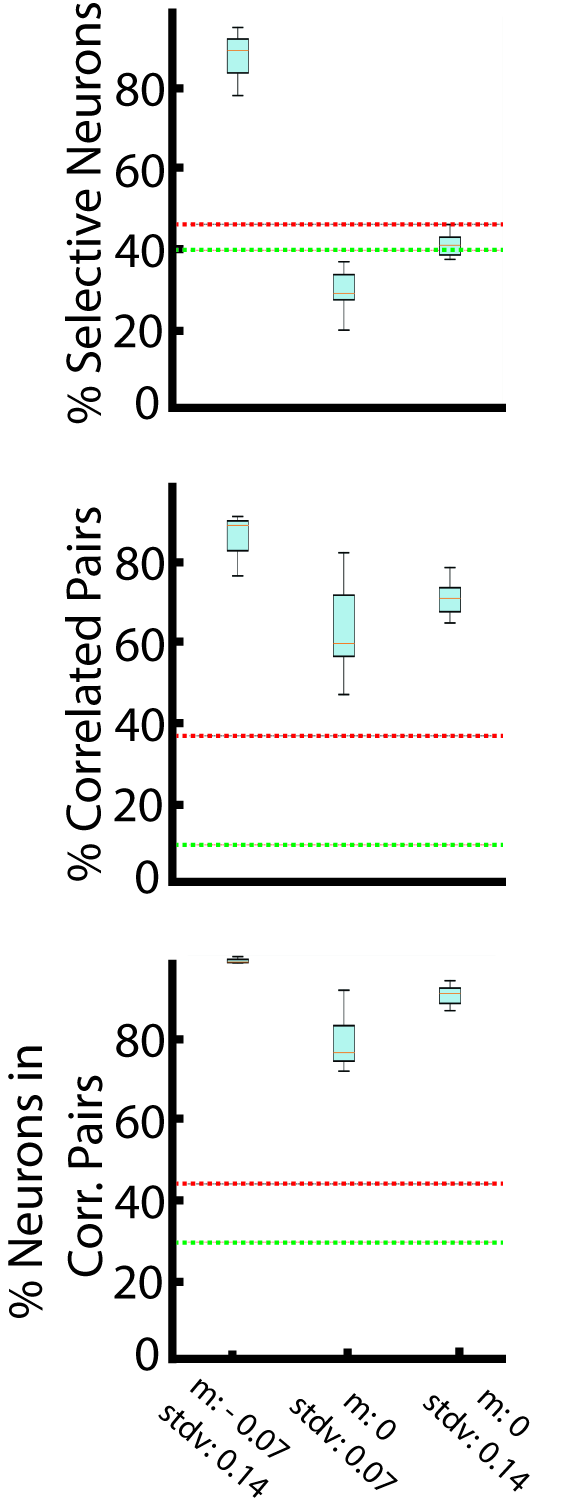

Supplement: S2 Fig — The middle column shows the parameters for random connectivity used in the rest of the manuscript (M: 0; STDV: 0.07). Left column shows results for connectivity with mean of -0.07 and STDV of 0.14, while the right column shows a network with mean 0 and STDV of 0.14. Networks with positive means did not learn the task. (TIF) [file pcbi.1012867.s004.tif]
