# Supplementary material for: Mixed recurrent connectivity in primate prefrontal cortex
Source: PLoS Comput Biol. 2025 Mar 11;21(3):e1012867. doi: 10.1371/journal.pcbi.1012867 (PMC11918408; doi:10.1371/journal.pcbi.1012867)
Supplement: Attachment — Submitted filename: Sigalas Response to Reviewers 2.docx [file pcbi.1012867.s007.docx]

**RESPONSES**

**Additional Editor Comments:**

As you will in the reviewers' comments, one of the reviewer is largely happy with the revision but most of the concerns raised by the second reviewer have remained unresolved. We therefore invite you one more time to adequtaly address the concerns of the reviewer. As I see it his/her comments are related to the robustness of the results and rigor in the approach -- we of course cannot compromise on that.

Thank you for giving us a chance to address the reviewer’s comments. As our answers below show, if we understand the reviewer’s concerns correctly, they arise from a misunderstanding of our goals and assumptions. Hopefully, the clarifications below show that our results are robust and rigorous.

**Reviewers' comments:**

**Reviewer #1:** The authors have improved the manuscript by providing a more detailed description of the model, a control regarding the overall connection strength and an extended discussion. While I appreciate these improvements, I also observe that my main critique has not been addressed: Their central claim ("the FEF, has a higher percentage of bump attractor connectivity (~20%) than […] the DLPFC", line 222-223) is still not supported by the data they present. What the authors show is that a model initialized with a higher percentage of bump attractor connectivity qualitatively reproduces spike train statistics from FEF better compared to a model with a lower percentage, which in turn better reproduces spike train statistics from DLPFC. No evidence for a causal relation between the models and the data has been shown. In the review of the first version, I suggested that (limited) evidence for such a relation could result from a rigorous set of controls that attempt to rule out any alternative explanation for the different model behavior except the percentage of bump attractor connectivity. The one single control the authors added is not remotely sufficient to tighten the evidence of causality. The fact that the models only loosely reflect the spike train statistics (as clarified in the author's responses) further weakens this evidence. The added methodical details on the model also allow for a more confident assessment that the necessary controls are next to impossible due to the large number of parameters and a lack of a clear theoretical foundation about the mechanisms underlying the model behavior.

We would like to thank the reviewer for pointing out an apparent misunderstanding regarding the main takeaway of our manuscript. It seems the reviewer interpreted our goal as proving, without any prior assumptions, that the bump attractor architecture directly causes PFC activity. We agree that demonstrating this would require comparing our findings—based on the bump attractor architecture—with all known (and even unknown) alternative architectures. **However, we want to clarify that this is not our goal**.

**Our starting assumption is that the bump attractor architecture exists in the primate PFC**. This assumption is supported by previous studies indicating that the bump attractor model aligns with the physiological properties of primate PFC activity (e.g., Compte et al., 2000; Wimmer et al., 2014; Parthasarathy et al., 2019; Barbosa et al., 2020; Stroud et al., 2023). For example, Wimmer et al. (2014) demonstrated that PFC activity exhibited drift patterns consistent with the bump attractor model, which in turn affected the monkeys' working memory recollection. In addition, Parthasarathy et al. (2019) showed that PFC activity contains a stable subspace for working memory information, further supporting the bump attractor model. In contrast, randomly initialized models trained on the same tasks produce dynamic codes that do not resemble the drifts and stable codes observed in the PFC (see Figure R1). These and other studies **provide evidence for a causal relationship between the bump attractor model and the primate PFC data.**

**Figure R1**. *Cross-temporal decoding of randomly-intiated RNN model (0% bump initialization). Dynamic coding is observed throughout the task (higher decoding performance in the diagonal compared to the off-diagonal)*

In contrast, and just as an example of models that we did not consider in our analysis, we do not assume that synfire architecture exists in the primate PFC, as there is no evidence supporting this from the literature. Thus, assuming that the primate PFC contains bump attractor connectivity, our goal is to assess the prevalence of this connectivity across different prefrontal regions. **We believe this provides a solid theoretical foundation for understanding the mechanisms underlying the behavior we modeled.**

In response to the first review, we carried out controls showing that randomly initialized networks with varying mean connectivities and standard deviations did not replicate the observed data properties (a control suggested by the reviewer). While the reviewer recommended exploring short-term plasticity, we opted to address this in the study limitations, as the multitude of possible models—given the various combinations of short-term plasticity parameters (length, strength, variance, etc.)—would result in a combinatorial explosion of models, as the reviewer acknowledged. Thus, we feel we adequately addressed the concerns raised in the first round.

If our clarification regarding the manuscript's goal does not alleviate the reviewer’s concerns, it may indicate that we are misunderstanding the nature of those concerns. In that case, we would appreciate it if the reviewer could specify what would be considered sufficient evidence to support our conclusions.

For reference, please see the conclusions summarized in the first paragraph of the discussion:

“*There is evidence that the bump attractor connectivity is found in the lateral prefrontal networks (Wimmer et al., 2014). However, an outstanding question is how much of this connectivity is present in different prefrontal regions. Here, we show that a posterior prefrontal region, the FEF, has a higher percentage of bump attractor connectivity (~20%) than a more anterior prefrontal region, the DLPFC (~7.5%).* .”

**References**

A. Compte, N. Brunel, P. S. Goldman-Rakic, X. J. Wang, Synaptic mechanisms and network dynamics underlying spatial working memory in a cortical network model. Cerebral Cortex 10, 910–923 (2000).

K. Wimmer, D. Q. Nykamp, C. Constantinidis, A. Compte, Bump attractor dynamics in prefrontal cortex explains behavioral precision in spatial working memory. Nat. Neurosci. 17, 431–439 (2014).

A. Parthasarathy et al., Time-invariant working memory representations in the presence of code-morphing in the lateral prefrontal cortex. Nat. Commun. 10, 4995 (2019).

J. Barbosa et al., Interplay between persistent activity and activity-silent dynamics in the prefrontal cortex underlies serial biases in working memory. Nat. Neurosci. 23, 16–18 (2020).

Stroud, Jake P., et al. "Optimal information loading into working memory explains dynamic coding in the prefrontal cortex." Proceedings of the National Academy of Sciences 120.48 (2023): e2307991120.

As a final remark, the authors claim in the beginning of the discussion that, to their knowledge, "this is the first study to attempt to infer network connectivity based on physiological properties" (line 225). By "physiological properties", I assume they mean the correlation and Fano factor of the spike trains. However, there is in fact a vast literature that attempted such inference. As a starting point, see de Abril, I. M., Yoshimoto, J., & Doya, K. (2018) for a review.

We agree with the reviewer that this statement is misleading. The point we were trying to make in the first submission related to the *architecture* of the network rather than the connectivity. In the revised version, we removed the term “architecture” from the manuscript based on Reviewer 2’s feedback. It was our oversight to fail to update this phrase. Indeed, several studies have attempted to infer connectivity between neurons using physiological measures (e.g., the simplest cases are studies that use pairwise neuronal correlation to infer pairwise neuron connectivity). This, and more complicated cases, are those covered by Abril et al. (2018) review. However, an inferred connectivity does not lead to an inference of the architecture (although the architectural information is embedded in the connectivity matrix). For example, if we shuffled the order of the units in the connectivity matrices of our mixed networks we would have the accurate connectivity of the network without insights about the underlying architecture: that there is a mixture of random- and bump attractor architectures (see Figure R2 below for an example).

Given that this phrase was misleading, we have decided to remove it from the manuscript.

**Figure R2.** ***(Left)*** *Connectivity matrix of a network initialized with 20% bump connectivity (post-training), sorted by initialization (bottom-right shows the units initialized with bump connectivity.* ***(Right)*** *Same network as in the left plot, but where the order of the neurons are shuffled. Both connectivity matrices are accurate, but the right one obscures the underlying bump connectivity.*

Given the lack of perspective to find a sensible set of controls, of theoretical foundation and of foundation in the literature, I regret that I have to recommend the rejection of the manuscript.

We hope the responses above convince the reviewer that these controls are unnecessary to reach our conclusions and that our approach rests on a solid theoretical foundation based on prior literature.

**Reviewer #2:** The formulation "... because certain properties of bump attractor networks are not shared by PFC neurons." compares network architecture with neuron properties, strictly speaking, which is unfortunate. Should change last part to "... shared by the PFC." or something equivalent.

Thank you for pointing this out. We have amended this error.

The sentence "There is also some an overlap between ...", "an " should be removed.

This phrase is not present in the revised version. It reads, “*There is also an overlap…*”
