# Supplementary material for: Mixed recurrent connectivity in primate prefrontal cortex
Source: PLoS Comput Biol. 2025 Mar 11;21(3):e1012867. doi: 10.1371/journal.pcbi.1012867 (PMC11918408; doi:10.1371/journal.pcbi.1012867)
Supplement: Attachment — Submitted filename: Response to reviewers.docx [file pcbi.1012867.s006.docx]

**Synthesis**

The manuscript was reviewed by two reviewers. Both found the paper very interesting. However, they have raised several concerns that need to be addressed in a major revision of the manuscript. Here are the main concerns:

The manuscript is quite sloppy and unclear in the description of the methods.

The locally of connectivity as required by the bumpNN is may not be consistent with the experimental data therefore it is important to discuss how BumpNN maps on the known cortical connectivity.

Related to the previous point, some of the key controls necessary to establish BumpNN style connectivity are missing in the current version of the manuscript. The reviewers have made some suggestions in this regard.

Detailed comments from the reviewers are appended to help you revise and prepare a point by point reply. We look forward to reading the revised manuscript.

Thank you for the comments. We have addressed all the reviewers’ comments, and now the manuscript has improved significantly. We hope that the changes are to the reviewers’ and editor’s satisfaction.

**Reviewer #1**

The authors attempt to test the potential existence of a bump attractor architecture in two regions of the primate prefrontal cortex (FEF and dlPFC) by matching several properties of electrophysiological activity in vivo with a recurrent neuronal network (RNN) trained to perform the same task as the monkeys. The network contains varying fractions of bump attractors (as compared to random connections without spatial structures) and is subject to varying degrees of external noise. These two parameters are varied to match the properties of interest. The authors find that activity in the FEF is consistent with an RNN with a higher percentage (20%) of bump attractors compared to dlPFC (7.5). They further compare decoding properties between models and data and find a number of similarities.

The author's approach to use computational models as a tool to uncover structures in experimental data is promising and timely. Also, the existence of attractor structures in the prefrontal cortex is a topic of intense debate currently, and this paper has the potential to contribute to this debate. However, in the current form, the analyses presented does not allow any valid statement about the existence of bump attractors in the prefrontal cortex. This is mainly due to the lack of proper controls: While properties such as increased noise correlations could indeed stem from a bump attractor synaptic architecture, there are many alternative explanations that cannot be excluded in the current research design. The simplest of these alternative explanations would be an increased level of overall connectivity (number of connections and/or synaptic weights) in the bump attractor architecture compared to the random connection pattern. In principle, evidence for the existence of bump attractors would require exhausting every possible (or at least, sensible) combination of parameters for random connectivity that may affect the correlation pattern, number of selective neurons, and so on. Only if neither of these combinations would yield a satisfactory fit to the data, one could conclude that a purely random connectivity pattern is insufficient to explain the observed data. Even in this case, a better fit with a bump attractor architecture would not exclude any other, non-random, pattern of connectivity that would explain the data as well (e.g. other models of working memory such as models based on short-term synaptic plasticity). But from such an analysis, one could at least conclude that the bump attractor architecture is superior in explaining the data over a random connection pattern - different from the study in the current form.

We thank the reviewer for allowing us to clarify our study's conclusions better. As the reviewer points out, increasing the overall level of connectivity leads to increased correlations. However, in our study, the overall connectivity of the bump-attractor and random connectivities were kept identical (mean of zero). That being said, to further explore the role of connectivity statistics in purely randomly initialized networks, we trained additional networks with different overall mean connectivity weight and standard deviations. We found that many of the networks never learned (i.e., networks with positive mean weights), but the ones that did learn deviated significantly from our brain-derived constraints. Thus, even though the bump attractor connectivity is one of the solutions that a random network could potentially learn, this solution does not appear to be reached regularly (if at all) in randomly initialized networks.

We added this to the manuscript results:

L177: “**We could not find models that matched the physiological properties of the data by training purely random models (0% bump attractor connectivity)(Figure S2).**”

***Figure S2. Purely random models (0% bump attractor architecture) failed to match the physiological constraints.*** *The middle column shows the parameters for random connectivity used in the rest of the manuscript (M: 0; STDV: 0.07). Left column shows results for connectivity with mean of -0.07 and STDV of 0.14, while the right column shows a network with mean 0 and STDV of 0.14. Networks with positive means did not learn the task.*

In the last paragraph of the discussion we provide limitations of our conclusions in line with what the reviewer is suggesting:

“Regarding the models, they contain several simplifications and assumptions that may influence the precise percentages of bump attractor connectivity that we estimated. First, the non-bump attractor network was modeled as a randomly connected network. However, randomness in the connections is not a necessary feature since other connectivities may coexist with the bump attractor connectivity. Second, we did not include short-term plasticity in the model units, which may be relevant for the encoding of working memory (Masse et al., 2019; Miller et al., 2018; Seeholzer et al., 2019; Tao & Libedinsky, 2024). Third, FEF and DLPFC are interconnected networks, but we modeled them separately. Multi-region models may allow better estimates of the proportion of bump attractors in these networks (Perich & Rajan, 2020). Fourth, we modeled each region as having 1 attractor network, while it is possible that each region has multiple attractor sub-networks (Wang & Kang, 2022). Thus, the precise values proposed (20% in FEF and 7.5% in DLPFC) should be taken as initial estimates of these regions’ recurrent connectivity.”

Apart from this main point, I noticed that many of the methodical details of the model are not well explained, and some choices may lead to problems in the analysis. In particular:

- According to the first equation in the methods section, there are no connections between the bumps and the random network, although this is implied in the text and Figure 1.

Thank you for pointing out this oversight. We have modified the description to clarify that the input u includes the activity of the other group of neurons along with the stimulus input. We have also modified Figure 7 to indicate what are the W_in_^Bump^ , W_in_^NonBump^ , W_rec_^Bump^, W_rec_^NonBump^.

“**where u, x, and z are the input, recurrent state, and output vectors. W_in_, W_rec_, and W_out_ are the input, recurrent, and output synaptic weight matrices. The input u includes the stimulus as well as the responses of the other group of units**.”

- The non-linear activation function needs to be specified.

We have included it in the latest version of the manuscript.

- The details of the two network architectures are missing, although potentially crucial to the results. In particular, all equations and parameters need to be included in the Method section.

We include all the model parameters in the “Model parameters” and “Training parameters” sections in the Methods Section. We also added an explanation of the additive stimulus noise (Behavioral Tasks section), which is constant for all the models. If the reviewer considers that some description is missing, could we request a clarification of what are the missing parts?

- Stitching parts of the time series together may cause problems when estimating correlations without further precautions, as it tends to induce non-stationarities, see Quiroga-Lombard et al., 2013, J Neurophysiol.

We thank the reviewer for bringing this point up. In our study we used neural data during the delay period, which is a stable period without changing stimuli nor changing cognitive demands. Furthermore, this activity was z-scored per location, thus we are removing a large source of possible non-stationarities in our data before running the correlation analyses. Perhaps some global sources of non-stationarity may exist (such as fluctuations of alertness), but it would be strange if these sources only affected a small proportion of neurons in both regions (i.e., only those neurons that show significant correlations), and that these sources would be different across brain regions, since we found very few neurons correlated across regions.

However, to explicitly discuss this concern, we have added the following section to the discussion:

“**It is important highlight potential issues with the analysis and the model selection. It is possible that non-stationarities in our data affected our cross-correlation estimation (Quiroga-Lombard et al., 2013). We minimized non-stationarities by using neural data during the delay period, which does not contain any changing stimuli nor changing cognitive demands. Furthermore, this activity was z-scored per location. The possibility remains that some global sources of non-stationarity may exist (such as fluctuations of alertness), which would affect our results. However, for this to be the case, we would need these global sources of non-stationarity to affect single neurons in each area differently, and also with different temporal profiles in FEF and LPFC, since we found very few neurons correlated across regions, which seems unlikely**. ”

- Key methodical concepts as RNNs and decoding properties need to be briefly explained in the Results section.

We have taken this comment into consideration and have made additions to the manuscript to address it:

Introduction: “**Recurrent neural networks (RNN) are networks that contain units that can mutually influence each other (i.e., unit A may influence – directly or indirectly - the activity of unit B, while unit B may also influence the activity of unit A)**.”

Results: “To determine whether these identified mixed models matched the population decoding properties of FEF and DLPFC, we performed cross-temporal decoding on the neural data and their corresponding models (Figure 5A). **We quantified two decoding properties: (1) the amount of decodable information, which can be measured with a time-specific decoder, and (2) the stability of the code used to encode this information, which can be measured by how well a decoder trained at one time point generalizes to another time point (Stokes et al., 2013)**”

- The rationale behind the chosen decoding properties is unclear to me..

We selected properties that are normally assessed in decoding studies, which are amount of decodable information (which can be assessed with a time-specific decoder, as we did here) and the stability of the code used to encode this information (how well a decoder trained in one time point generalizes to another time point). We now include a citation of one of the earliest studies to use this analysis in neural signals to justify our selection (Stokes et al., 2013).

Results: “To determine whether these identified mixed models matched the population decoding properties of FEF and DLPFC, we performed cross-temporal decoding on the neural data and their corresponding models (Figure 5A). **We quantified two decoding properties: (1) the amount of decodable information, which can be measured with a time-specific decoder, and (2) the stability of the code used to encode this information, which can be measured by how well a decoder trained at one time point generalizes to another time point (Stokes et al., 2013).**”

- The precise criteria for match between model and data for each of the properties under investigation are not mentioned. It seems that there is only a partial match for some of them.

We did not apply any specific criteria since we were just looking for a qualitative match between the properties. These are specified in the results section:

“In both regions and models, neurons/units forming part of one or more significantly correlated pairs (top plots in Figure 5A) had more decodable target information (p < 0.001; Figure 5B). Furthermore, the generalizability of the memory code during the first 500 ms of the Delay 1 period was higher in DLPFC and the 7.5% model compared to that found in FEF and the 20% model (p < 0.001; Figure 5C).

- Figure 5A shows only little similarity between the decoding properties of the data and the corresponding model. This needs to be discussed.

We presume that the reviewer refers to the higher overall decoding performance in the models. This is true, and now we added a section to the results to highlight this difference:

Results: “**One difference between the brain regions and their corresponding models is that the overall decoding performance in the models was higher than in the brain regions, suggesting that some properties of the neural data are not captured by the models**.”

Differences are to be expected, since, as discussed in the last sentence of the Results section we did not fit the models to match the data:

“It is important to emphasize that these models (20% and 7.5%) were only selected based on single neuron and pairwise correlation data, so the observation that their population decoding behavior matches that of the data provides independent support for the proposed models”.

We have added a discussion of this point in the discussion section:

“**One of the most clear differences between our models and the PFC data is that the decoding of working memory information in the models generally exceeded that of the brain data (Figure 5). We speculate that this is the result of an underestimation of the noise level in the models. We explored different levels of noise in the models, and settled on a level that allowed us to match all the constraints derived from the data, including the fano factor. However, the fano factor of rate networks, like the ones we employed for our models, may not correspond perfectly to the fano factor of spiking networks, like the ones recorded from the PFC regions, because we are not considering the variability induced by the stochasticity of spike generation (Xie et al., 2022). Thus, we speculate that a more accurate estimate of PFC connectivity could be achieved with spiking networks instead of rate networks.**”

As a final point, the discussion section seems very brief and somewhat superficial to me. I would have expected a discussion of the relation of the current research to similar studies in the past and a more thorough reflection on the implications of the findings and, in particular, the methodical limitations.

We thank the reviewer for their opinion on the discussion section. As far as we know there are no similar studies in the literature that attempt to infer connectivity properties based on physiological properties. We have added a statement in the discussion to reflect this:

“**As far as we know, this is the first study to attempt to infer network connectivity based on physiological properties.**”

We have also added several new sections to the discussion which we hope will reflect a deeper treatment of the topic:

“**The existence of bump attractor connectivity in the PFC has been criticized because certain properties of bump attractor networks are not shared by PFC neurons. For example, the PFC does not contain an orderly organization of neurons with the precise connectivity required by the bump attractor model (Constantinidis et al., 2001; Leavitt et al., 2018). However, a recent study showed that PFC neurons with similar spatial or shape selectivity are more likely than chance to be encountered at short distances from each other (Sun et al., 2024), providing a possible solution to this line of criticism. Furthermore, the bump-attractor connectivity does not need to have cell bodies localized in close proximity. Rather, all that is needed is for neurons, wherever they are localized, to receive structured inputs and connect to each other in a structured way (the bump-attractor connectivity).**

**An additional criticism is that the selectivity of PFC neurons is not always consistent between stimulus and delay periods, while that is the case for bump attractor networks (Rao et al., 1999; Spaak et al., 2017). Our results, which suggest that the bump-attractor connectivity is only present in a small percentage of neurons, could reconcile the observation that some PFC neurons maintain the selectivity between stimulus and delay, but the majority do not (Rao et al., 1999; Spaak et al., 2017). We should emphasize that we are not claiming that the canonical bump-attractor connectivity is present in the PFC, since our initialized bump-attractor connectivity is modified after training. What we are claiming is that a connectivity resembling the bump-attractor (with units with similar selectivity exciting each other and inhibiting units with dissimilar connectivity) is present in different proportions in FEF and DLPFC.**

**One of the most clear differences between our models and the PFC data is that the decoding of working memory information in the models generally exceeded that of the brain data (Figure 5). We speculate that this is the result of an underestimation of the noise level in the models. We explored different levels of noise in the models, and settled on a level that allowed us to match all the constraints derived from the data, including the fano factor. However, the fano factor of rate networks, like the ones we employed for our models, may not correspond perfectly to the fano factor of spiking networks, like the ones recorded from the PFC regions, because we are not considering the variability induced by the stochasticity of spike generation (Xie et al., 2022). Thus, we speculate that a more accurate estimate of PFC connectivity could be achieved with spiking networks instead of rate networks**.”

**Reviewer #2**

Review report for the manuscript entitled "Mixed recurrent
connectivity architecture in primate prefrontal cortex" by Sigalas and
Libedinsiky
====================================================
In this manuscript the authors aim to estimate the degree of recurrent "bump attractor network" architecture (BumpNN) in the DLPFC and FEF of thee non-human primates while performing a visual delayed match to sample working memory task. The method to do this relies on noise correlation between selectively active neurons and network units respectively. The network model used was a recurrent neural network with a bump attractor ("ring") network embedded in a matrix of randomly connected units, which also connected reciprocally to the BumpNN network. By matchin the experimentally measured noise correlation and other measures of activity between selectively active PFC neurons and those of the BumpNN units with similar response properties (and thus connected) they estimates that the degree of BumpNN-ness was 7.5 and 20 %.

Major comments
----------------------
Generally, I found this paper quite interesting, relevant and quite well written, though somewhat sloppy and unclear when it comes to e.g. detailed model descriptions. Some suggestions follow in the Minor comments section. I have one major concern though, and this relates to the non-critical presentation of BumpNN as a "conspicuous" model of PFC. I think it can have some relevance, but the way to map such an architecture to neocortex is by no means obvious. A main criticism has been that the local representation of the memory of an experimentally stimulated position in the field of view predicted by the model, i.e. a local connected and reverberating group of neurons goes against what many experimental data and computational models of distributed memory predicts. However, it is rather easy to get around this by emphasizing not the geometrical locality but the mutual connectivity between the units in the BumpNN. If the neurons in the cortical "BumpNN" is instead distributed over a larger cortical area but with the same connectivity, the dynamics and noise correlations would likely be quite similar and the results of the paper still valid. For discussion see e.g. Neural Mechanisms of Working Memory Accuracy Revealed by Recurrent Neural Networks by Xie, Liu, Constantinidis and Zhou, Frontiers 2022 (doi: 10.3389/fnsys.2022.760864).
What the architecture in the real neocortex actually looks like is still not clear, so I request addition of this alternative view in a prominent place in the manuscript, or alternatively a more detailed explanation how to map the BumpNN to neocortical architecture.

We thank the reviewer for this insightful comment. We agree with the point raised, so we have added statement in the second paragraph of the discussion to clearly emphasize this point:

“**The existence of bump attractor connectivity in the PFC has been criticized because certain properties of bump attractor networks are not shared by PFC neurons. For example, the PFC does not contain an orderly organization of neurons with the precise connectivity required by the bump attractor model (Constantinidis et al., 2001; Leavitt et al., 2018). However, a recent study showed that PFC neurons with similar spatial or shape selectivity are more likely than chance to be encountered at short distances from each other (Sun et al., 2024), providing a possible solution to this line of criticism. Furthermore, the bump-attractor connectivity does not need to have cell bodies localized in close proximity. Rather, all that is needed is for neurons, wherever they are localized, to receive structured inputs and connect to each other in a structured way (the bump-attractor connectivity).** ”

I would recommend the term "bump attractor connectivity" or "bump attractor properties" rather than "bump attractor architecture" at, for instance, the first line in the Discussion section and several other places. It will take some careful reformulations to make the text consistent in this regard.

We agree with the change, and have replaced the terms where appropriate.

Minor comments
----------------------
In the Methods section, the description of the RNN model with embedded BumpNN needs to be expanded and improved. It lacks citations and reference to standard RNN methodology. Is it etirely home cooked?

We used standard software (PsychRNN Ehrlich et al., 2021) and standard parameters (see references below for some examples). We have used the provided framework to extend the existing basic RNN classes to the mixed RNNs of bump and non-bump units as described in the manuscript.

Xie, Y., Liu, Y. H., Constantinidis, C., & Zhou, X. (2022). Neural mechanisms of working memory accuracy revealed by recurrent neural networks. *Frontiers in Systems Neuroscience*, *16*, 760864

Driscoll, L. N., Shenoy, K., & Sussillo, D. (2024). Flexible multitask computation in recurrent networks utilizes shared dynamical motifs. *Nature Neuroscience*, *27*(7), 1349-1363.

McMahan, B., Kleinman, M., & Kao, J. (2021). Learning rule influences recurrent network representations but not attractor structure in decision-making tasks. *Advances in Neural Information Processing Systems*, *34*, 21972-21983.

We have added this to the methods section:

L475: “**Network parameters were chosen based on prior studies (Driscoll et al., 2022; McMahan et al., 2021; Xie et al., 2022)**.”

1. It should be clearly stated that no learning is used but the BumpNN is entirely prewired.

Thank you for highlighting this. Our networks were only initialized with the BumpNN connectivity, but the whole network (including the BumpNN) was trained. We have clarified this point in the results section:

“To determine whether the results identified in the FEF and DLPFC are consistent with the bump attractor or the mixed model, we initialized mixed models with different percentages of bump attractor connectivity (5% to 100%, where 100% corresponds to a pure bump attractor connectivity), and then trained these RNNs to perform the same tasks as the monkeys **(the training modified the weights of all the units in the model, including the recurrent weights initialized with the bump attractor connectivity).**”

2. What is "intrinsic time scale" of the unit. Does it refer to their "membrane time constant" for instance. It seems too long (200 ms) for this, so what does it correspond to?

In our RNN model the time constant τ, specifies the rate of decay of the network state. When modeling large populations of neurons with RNNs what is important in the literature is the ratio between the constant τ and the timestep dt (dt/τ). A typical range is between ¼ to ⅕. In our case dt=50ms and τ=200ms (see the same references mentioned earlier).

3. Expressions like "near the diagonal", "mapped to a group of adjacent bump units", "also some overlap between groups of adjacent stimuli" need to be quantified to allow reproduction of the results.

This is a valid concern. Thank you for bringing it up. We have modified the manuscript to be more specific:

“**More specifically, the synaptic weights of each bump unit were given by the following equation adapted from (Wimmer et al., 2014):**

**Where κ=3 and θ∈[0, 2π]. The recurrent weight matrix of non-bump units does not have such a structure (Figure 7), and all the weights are randomly initialized.**”

We also modified the following section for clarity:

“The matrix W_in^((bump) ) has a spatial structure (Figure 7), so each target input stimulus is mapped to a group of **ten** adjacent bump units. **There is also some an overlap between these groups of size one at both ends of the group** ”

4. The "- Training Parameters:" subsection came as a surprise since the architecture is described as prewired. It should be clearly described that this network also have an output population and how it is connected and trained. This section should also be adjacent to the
other model subsections.

Thank you for giving us the change to clarify that again. As mentioned in the previous comment all our models are indeed trained to perform the Target-Distractor task. This is mentioned in the abstract, introduction and early in the results section. We have added the following to the results section to be more clear about this:

“To determine whether the results identified in the FEF and DLPFC are consistent with the bump attractor or the mixed model, we initialized mixed models with different percentages of bump attractor connectivity (5% to 100%, where 100% corresponds to a pure bump attractor connectivity), and then trained these RNNs to perform the same tasks as the monkeys **(the training modified the recurrent weights of all the units in the model, including the recurrent weights initialized with the bump attractor connectivity)**. ”

In the Training Parameters section we mention that we are freezing all the input Weights of the model and train the rest W_rec_^Bump^, W_rec_^NonBump,^ W_out_. We have made additions in the manuscript to explicitly state that the variable r in the last equation of the model where it is multiplied with the weights of the output population (W_out_r) is the concatenation of the r^bump^ and r^non-bump^ of each group of neurons from the previous equations. Finally we applied your feedback regarding the location of “Training Parameters” section and we moved it right after the model and model parameters sections.

“T**he variable r in equation (5) is the concatenation of r^bump^ and r^non-bump^ and represents the responses of all the units in the network**.”

5. The "scipy" module should at least be the "Python scipy module".

We modified that part in the manuscript to correctly address this comment.

6. The concept or low and high dimensionality of representations is used in many places. But these concepts are not defined but should be. An example definition is: "Linear dimensionality: the number of principal components required to account for a fixed proportion of variance in the data. For low-dimensional data, the variance is concentrated in the first few principal components with the largest eigenvalues. For high-dimensional data, the variance is distributed across many principal components with the eigenvalues of similar magnitude. Intuitively, the dimensionality corresponds to the extent of the linear subspace occupied by the data, or the number of separate patterns exhibited by the data." [TA Engel an NA Steinmetz (2019), New perspectives on dimensionality and variability from large-scale
cortical dynamics, Current Opinion in Neurobiology 58, 181-190.]

Thank you for pointing this out. We have made amendments to include the definition of these terms we are using in the manuscript:

“An important feature of recurrent neural network dynamics is the dimensionality of the network’s representations: **the number of principal components required to account for a fixed proportion of variance in the data (Engel & Steinmetz, 2019)**.”
