# Supplementary material for: Mixed recurrent connectivity in primate prefrontal cortex
Source: PLoS Comput Biol. 2025 Mar 11;21(3):e1012867. doi: 10.1371/journal.pcbi.1012867 (PMC11918408; doi:10.1371/journal.pcbi.1012867)
Supplement: Attachment — Submitted filename: Response_to_Reviewers_auresp_3.docx [file pcbi.1012867.s008.docx]

**Additional Editor Comments :**

Thanks for revising the manuscript. One of the reviewers is happy with the revision. However, concerns of one of the reviewers remain unaddressed. The reviewer makes a valid point. You essentially have explore only class of networks (i.e. the continuum of bum attractor type connectivity and random connectivity). Of course you will find that some combination of attractor type and random connectivity will be the best match for any given network. I do see value of the manuscript but the claims should be revised and it should be clearly stated that only one class of network were studied and another class of networks could have a better match. Therefore I invite you to revise the text once more and present the results in the right context.

Thank you for your comments. To address your (and the reviewer’s) comments, we have added the requested caveats in the introduction and discussion sections. We hope that these changes are sufficient to address the concerns:

Introduction

“*Recurrent neural networks (RNN) are networks that contain units that can mutually influence each other (i.e., unit A may influence – directly or indirectly - the activity of unit B, while unit B may also influence the activity of unit A). RNN models with attractor states have been shown to model many neural processes in the brain (Khona & Fiete, 2022). Specialized versions of such computational models (with local excitation and long-range inhibition), referred to as bump attractor models (Compte et al., 2000; Figure 1, left), can replicate several properties of prefrontal networks, suggesting that the bump attractor connectivity is present in these regions (Parthasarathy et al., 2019; Wimmer et al., 2014).* ***While other model classes may also be present in the PFC, we are only aware of experimental evidence supporting the presence of bump-attractor networks in the PFC. Thus, in this study we focus on this model class****.”*

Discussion

L296: “*It is important highlight potential issues with the analysis and the model selection.* ***Firstly, we assume that the bump-attractor connectivity is present in the PFC, which is only one network class out of the many possible ones. While this assumption is supported by indirect empirical evidence, it is plausible that it is wrong, and a different class of networks could lead to a better match with the data****.* ”

**Journal Requirements:**

1) We have noticed that you have a list of Supporting Information legends for Supplementary tables in your manuscript. However, there are no corresponding files uploaded to the submission. Please upload them as separate files with the item type 'Supporting Information'.

We have uploaded the files now.

2) Please provide a complete Data Availability statement in the online submission form.

We have completed it now.

**Reviewers' comments:**

Reviewer's Responses to Questions

Reviewer #1: I appreciate the clarification the authors made. I was aware that they did not aim to test for the existence of bump attractors in the cortex. However, this does not change my impression that the study suffers from a basic logical flaw: The authors show that a certain feature of a particular model, namely the degree of bump attractor connectivity, changes the quality of fit to data from two parts of the cortex. From that, they infer that the difference in bump attractor connectivity must also exist between the two parts of the cortex. This inference would only be valid if one could show that the change in bump attractor connectivity is not only sufficient, but also necessary to explain the difference in the fit to the two data sets. As already discussed, this is not the case: There are many other parameters that could affect the model, potentially in the same way as bump attractor connectivity. I offered one control which the authors indeed conducted, but I did not suggest that this single control was sufficient. Rather, I agree with the authors that investigating all thinkable controls would be infeasible. However, different from the authors, I conclude that the proposed inference is infeasible in general, at least while a more thorough theoretical framework is missing.

We would like to thank the reviewer for clarifying their concern. We have been careful in our interpretation of the results to avoid claiming that our results “prove” anything, but rather, they are suggestive, given the prior literature and the results presented. We have also added an explicit caveat to the discussion to expose the reviewer’s concern:

L295: “*It is important highlight potential issues with the analysis and the model selection.* ***Firstly, we assume that the bump-attractor connectivity is present in the PFC, which is only one network class out of the many possible ones. While this assumption is supported by indirect empirical evidence, it is plausible that it is wrong, and a different class of networks could lead to a better match with the data.***”

And a similar caveat in the introduction:

“*Recurrent neural networks (RNN) are networks that contain units that can mutually influence each other (i.e., unit A may influence – directly or indirectly - the activity of unit B, while unit B may also influence the activity of unit A). RNN models with attractor states have been shown to model many neural processes in the brain (Khona & Fiete, 2022). Specialized versions of such computational models (with local excitation and long-range inhibition), referred to as bump attractor models (Compte et al., 2000; Figure 1, left), can replicate several properties of prefrontal networks, suggesting that the bump attractor connectivity is present in these regions (Parthasarathy et al., 2019; Wimmer et al., 2014).* ***While other model classes may also be present in the PFC, we are only aware of experimental evidence supporting the presence of bump-attractor networks in the PFC. Thus, in this study we focus on this model class****.”*

If the authors could establish that the investigated activity signatures should be affected by bump attractor connectivity just in the proposed way, the (not too strong, by the way) empirical evidence could be seen as a confirmation of the theory.

The results presented in Figure 5 attempt to show exactly what the reviewer is asking for. We provide evidence that activity signatures that differ between the 2 regions naturally emerge from the proposed models (even though the models were not selected based on these activity signatures:

L204: “*It is important to emphasize that these models (20% and 7.5%) were only selected based on single neuron and pairwise correlation data, so the observation that their population decoding behavior matches that of the data provides independent support for the proposed models*”

As it stands, the proposed difference between the two brain regions in bump attractor connectivity is merely a moderately supported hypothesis, which could be the starting point of a manuscript, but is, in my mind, insufficient for a full publication in PLoS Computational Biology. I regret that I cannot come to a more positive conclusion, while the final decision is of course with the editor.

Reviewer #2: I accept the revision done.
